# Chicago Medical Society

**Published:** 1886-12

**Authors:** 


### Society Reports.

### CHICAGO MEDICAL SOCIETY.

Officially Reported for The Atlanta Medical and Surgical Journal.

Stated Meeting, October 18, 1886.

The President, E. J. Doering, M. D., in the chair.

Dr. R. W. Bishop read a paper entitled

### "IS ALOPECIA PREMATURA CONTAGIOUS?"

Dr Bishop thought the disease due to micro-organisms upon the shafts of the hair, and that it is contagious. He has made a series of experiments, assisted by Dr. Oscar Lassar. A typical case was that of a perfectly healthy young man whose head was nearly bald on top. The hair from the diseased surface was brittle and came out easily when pulled. Microscopic examination revealed a large number of fungi on the scalp and the shafts of the hair, the root being free from the parasite. The diseased hairs were cut and mixed with vaseline, which was rubbed on the skin of healthy rabbits, and in two weeks the hair entirely disappeared from the parts which had been rubbed. Experiments were continued, and it was found that the hairs from the inoculated animals possessed increased virulence.

The patient was treated as follows: The head was thoroughly washed for fifteen minutes with tar soap, which was removed with warm water. The head was then exposed to a warm water douche, which was gradually cooled until the water was quite cold; it was then rubbed with a rough towel until dry, and afterwards washed with a solution of corrosive sublimate 1:500. This was removed and a 1-2 per cent. of lithol applied and, after this, 1 1-2 per cent. carbolic oil was applied very slowly. The treatment was continued daily for eight weeks, and the result was a fine growth of new hair with beginning pigmentation at the end of three months.

Dr. Joseph Zeisler thought this was a disease which possibly might be produced by vegetable organisms. He knew of Dr. Bishop's experiments, but still there were strong objections to the value of these experiments. Michelson made some experiments by using a mixture of vaseline and rancid oil and rubbing it on the skin of guinea-pigs, and after these inunctions the animals got bald on the places anointed, although neither sick hairs nor scales were used. There are older experiments which show that animals fed on old cheese or hard-boiled egg get bald. Another point was that the disease is so frequently met with in men and so rarely in women, between whom there are plenty of chances for contagion. It is an ascertained fact that the disease most frequently occurs in men who in their earlier years (17 to 25) suffer from pityriasis of the scalp, so that there certainly is a causal relation between this affection and alopecia. After all, he thinks that the contagiousness of this disease is still an open question.

Dr. Frank Billings spoke on

HOSPITAL PRACTICE IN VIENNA WITH EXHIBITION OF NEW URINE TESTS, NEW INSTRUMENTS, ETC.

The hospital at Vienna contains about 2,500 beds. The number of deaths per year is about 3,000. Prof. Nothnagel, who presides over the first medical clinic, has from 80 to 100 patients in his ward continuously. Histories of the patients are written by assistants and left in charge of the nurses. Temperature charts are kept of important cases, the temperature being taken every two hours. Daily clinical and microscopical examinations are made of the urine in all important and grave cases. Dr. Billings gave an illustrated description of the tests employed. He said that in the Vienna hospitals some form of tuberculosis is found in nearly 70 per cent. of the deaths. The treatment of acute diseases is generally expectant, and in typhoid fever, milk and other liquid diet is used. Previous to 1879, when the city obtained its water from the Danube River, typhoid fever was almost epidemic in Vienna. In that year they put up new water-works, and since that time not one case has developed in the city. The obstetrical department is divided into three clinics, with 3,000 confinements in each clinic yearly. Four cases of sublimate poisoning occurred last winter. The autopsies showed ulceration throughout the alimentary tract with charred, black appearance of the mucous membrane throughout the colon and rectum. The solution used in these cases was 1 to 4,000, bichloride of mercury. Abortion is treated by rest in bed with non-interference, unless too severe hemorrhage occurs, when tampons are used. In the gynecological department Prof. Braun performs laparotomy every Wednesday. Where a part of tumor is left in the stump, the prognosis is bad, even when treated externally, because of the low vitality of the tumor tissue, which becomes necrotic. In operations about the interior vaginal wall no anæsthetic is used. In the surgical wards of Profs. Billroth and Albert, closest attention is paid to cleanliness. The operating rooms are constructed with floors inclined to the centre, where a grating allows all blood, water, etc., to flow away. The floor is thoroughly douched every day. Sponges, silk sutures and towels used in operations are boiled in a 5 per cent. solution of carbolic acid for one hour, then placed in a 5 per cent. solution for fourteen days. Cutting instruments are polished and placed in a 2½ per cent. solution of carbolic acid during the operation. Instruments used in operations for abscesses, etc., are heated in flame and sent to the instrument maker to be repolished. A 10 per cent. solution of carbolic acid is used for irrigating wounds during operation. For partial amputation of the tongue a bloodless method is used as follows: First, a double stout suture thread is passed through the centre of the tongue from below upward and backward, beginning at the frænum; the two threads are twisted and tied upon the side firmly enough to control the vessels. The part is then amputated smoothly by taking out a triangular section. Two deep and a sufficient number of superficial sutures close the wound after the vessels are secured. A bacteriological laboratory is connected with the pathological department, and cultivations of bacteria are made from typhoid fever, pneumonia, erysipelas, glanders, septicæmia and other diseases. Experiments with bacterial cultures are made upon lower animals. Nearly every department of the hospital now has a bacterial laboratory, and the search for new forms and confirmation of already discovered bacteria goes on with enthusiasm.

Dr. C. W. Purdy said that in testing the urine for evidence of kidney diseases, the only proteids of any significance were serum albumin and globulin. The presence in the urine of peptone, hemialbumose, etc., points to morbid conditions outside of the kidneys, and whatever light they may shed upon general conditions, they afford us no information whatever as to the state of the kidneys. He mentioned this because so much had been written of late on peptonuria, and the various transition proteids, that an impression seemed to have arisen that the presence of these in the urine was of scarcely less importance than that of serum albumin. The only bearing these non-homogeneous proteids have upon the subject is the fact that their occasional presence in the urine may, with certain tests, be mistaken for serum albumin, unless great care be exercised.

He believed the most delicate of all tests for albumin in the urine to be the potassia mercuric iodide with citric acid, but that it had not met with general favor thus far on account of the errors liable to arise through its use. It is necessary to discriminate between the precipitates formed by this test with peptone, alkaloids, above all with mucin, and that formed by serum albumin. It is true that heat clears up the precipitates due to peptones and vegetable alkaloids, but not so with mucin, the latter being practically indistinguishable from albumin. He has lately, however, come upon a method which he believes will correct not only the errors due to the presence of mucin, but also those likely to arise from the presence of alkaloids and peptone when precipitated by this test. The method is very simple, viz.: after having applied the reagent to the suspected urine, if a precipitate be formed, add hydrochloric acid in volume equal to the quantity of urine tested; if mucin, peptone or vegetable alkaloids be the cause of the turbidity, it promptly clears up; while if due to albumin the precipitate becomes flocculent and settles, but does not dissolve. A considerable number of experiments have shown him that hydrochloric acid in volume equal to one-half the quantity of urine tested quickly clears up the peptone, alkaloid and mucin precipitate, while it requires at least two volumes of hydrochloric acid to dissolve the slighter traces of albumin in urine when precipitated by the mercuric test.

With regard to sugar testing: In addition to the test brought forward by Dr. Billings, two new ones have recently been introduced, both of which are of such exceeding keenness that they are claimed to be able to detect 0.00001 per cent. of sugar. These tests are, alcoholic solutions 15 to 20 per cent. of alpha-naphthol and thymol. Two drops of either of these solutions are added to two cubic centimetres of urine, and the mixture briskly shaken; sulphuric acid is next added in quantity equal to the volume of urine and again briskly shaken. In the case of alpha-naphthol a deep violet color is developed in the presence of sugar, and dilution with water throws down a violet blue precipitate, soluble in alcohol with a yellow color, or in caustic potash with a deep yellow. In the case of thymol a dark red color is produced and, on adding water, a precipitate settles, which dissolves with alcohol, forming a yellow color more decided if ammonia be added.

Dr. Purdy often had samples of urine sent him, which, though loaded with albumin, no casts could be found therein. These were samples of urine which had been long passed—perhaps several days before, alkaline fermentation having occurred, and the urine rendered alkaline quickly dissolves the casts. In searching for renal casts it is of the greatest importance to have the urine as freshly passed as possible. His method of examining urine for casts is as follows: First, he prefers to have the urine passed at his office. If the urine be neutral or alkaline in reaction, he renders it frankly acid by the addition of dilute acetic acid. In all cases he adds a solution of resorcin to the urine, which prevents change for weeks. The urine thus treated is set aside in a conical glass, carefully covered and allowed to stand for from twenty-four to forty-eight hours; at the end of this time, a few drops—not more than ten—are taken up by a glass tube from the bottom of the glass and one or two drops placed upon a glass slide and examined under the microscope. He had had no difficulty in finding casts by this method if they were present in the urine even in sparce numbers.

Dr. Frank S. Johnson described a new form of *hæmoglobinometer*, viz.,

THE HÆMOTER OF VON FLEISCHL.

It consists of a stand with a horseshoe base, an upright, a stage and a well, divided perpendicularly in two equal compartments and closed below by thin glass. One-half of the well is to hold blood of known dilution; the other half is for clear water. This well fits an opening in the stage. Beneath the stage is a plane white reflector. On the under side of the stage is a frame that can be racked back and forth. Set in it is a narrow wedge-shaped piece of ruby glass whose width is one-half that of the opening in the stage. The thicker end of the wedge gives by transmitted light the color of a dilution of blood containing the maximum amount of hæmoglobin. The thinner portions correspond with the color of a dilution of blood poorer in hæmoglobin. The percentage of variation from the normal amount of hæmoglobin is estimated by comparing the color of the blood solution with some part of the ruby wedge. Only artificial light can be successfully used for the examination.

With the instrument are several capillary tubes for measuring the amount of blood to be used for comparison with the colored glass standard. The necessary amount of water for making the dilution is measured in one of the chambers of the well.

The blood for examination is best obtained by pricking the ball of an uncompressed finger and forcing out a drop by gentle pressure. The amount needed is taken in the capillary tube. One of the halves of the well having been previously half or two-thirds filled with water, the blood can be easily washed from the measuring tube into it. Then both halves of the well are accurately filled with water so that the upper surfaces are plane. A small pipette is furnished for this purpose. The well is then so adjusted that the half holding water is above the colored glass, and that holding the blood solution receives its light directly from the white reflector. The next step is to so adjust the ruby wedge that the light it transmits corresponds in color with that passing through the blood solution. The observer then reads off the percentage of the normal amount of hæmoglobin as indicated by a scale graven upon the metal frame carrying the glass wedge. The average percentage of hæmoglobin in the blood of healthy individuals varies greatly with age and sex. The average is from 12 to 13 per cent. The percentage of this amount is ascertained by comparison with the color scale. This result should be corrected as far as possible for the known variation of hæmoglobin in healthy blood. Taking as the normal percentage that found in the blood of healthy individuals between 25 and 30 years of age, it is found that in the first few weeks of life the percentage is greatly in excess; but after six months or a year, it is below the adopted standard, reaching it again at about 25 or 26 years of age, and that after the thirtieth year the amount is below the normal, but is variable.

The instrument recommends itself to the ordinary practitioner. It does not exactly replace the hæmocytometer, but in all cases where it is only necessary to watch from time to time the rise and fall of the hæmoglobin in the blood, it can be done much quicker and more accurately than by the hasty counting of the corpuscles by the microscope.

Dr. J. J. M. Angear thought that it is necessary to count the globules, because we have conditions where the red corpuscles are normal in number but deficient in hæmoglobin; hence the necessity of both instruments.

Dr. C. E. Webster, chairman of the Pathological Committee, exhibited a spinal column and said that it showed caries resulting in the entire destruction of the bodies of one of the vertebræ, without the protection of the characteristic deformity before the removal of the ligaments, no curvature being noticeable.

